# Effect of Pd_2_Spermine on Mice Brain-Liver Axis Metabolism Assessed by NMR Metabolomics

**DOI:** 10.3390/ijms232213773

**Published:** 2022-11-09

**Authors:** Tatiana J. Carneiro, Martin Vojtek, Salomé Gonçalves-Monteiro, Ana L. M. Batista de Carvalho, Maria Paula M. Marques, Carmen Diniz, Ana M. Gil

**Affiliations:** 1 Department of Chemistry, CICECO—Aveiro Institute of Materials, University of Aveiro, 3810-193 Aveiro, Portugal; 2 LAQV/REQUIMTE—Associated Laboratory for Green Chemistry of the Network of Chemistry and Technology, Laboratory of Pharmacology, Department of Drug Sciences, Faculty of Pharmacy, University of Porto, 4150-755 Porto, Portugal; 3 Molecular Physical-Chemistry R&D Unit, Department of Chemistry, University of Coimbra, 3004-535 Coimbra, Portugal; 4 Department of Life Sciences, Faculty of Science and Technology, University of Coimbra, 3000-456 Coimbra, Portugal

**Keywords:** palladium(II), platinum(II), spermine, Pd_2_Spm, cisplatin, mice, brain, liver, NMR, metabolomics

## Abstract

Cisplatin (cDDP)-based chemotherapy is often limited by severe deleterious effects (nephrotoxicity, hepatotoxicity and neurotoxicity). The polynuclear palladium(II) compound Pd_2_Spermine (Pd_2_Spm) has emerged as a potential alternative drug, with favorable pharmacokinetic/pharmacodynamic properties. This paper reports on a Nuclear Magnetic Resonance metabolomics study to (i) characterize the response of mice brain and liver to Pd_2_Spm, compared to cDDP, and (ii) correlate brain-liver metabolic variations. Multivariate and correlation analysis of the spectra of polar and lipophilic brain and liver extracts from an MDA-MB-231 cell-derived mouse model revealed a stronger impact of Pd_2_Spm on brain metabolome, compared to cDDP. This was expressed by changes in amino acids, inosine, cholate, pantothenate, fatty acids, phospholipids, among other compounds. Liver was less affected than brain, with cDDP inducing more metabolite changes. Results suggest that neither drug induces neuronal damage or inflammation, and that Pd_2_Spm seems to lead to enhanced brain anti-inflammatory and antioxidant mechanisms, regulation of brain bioactive metabolite pools and adaptability of cell membrane characteristics. The cDDP appears to induce higher extension of liver damage and an enhanced need for liver regeneration processes. This work demonstrates the usefulness of untargeted metabolomics in evaluating drug impact on multiple organs, while confirming Pd_2_Spm as a promising replacement of cDDP.

## 1. Introduction

Polynuclear chelates of palladium(II) with biogenic polyamines (e.g., putrescine, spermidine or spermine) have displayed promising antitumor properties, emerging as possible alternatives to the clinically used mononuclear platinum(II)-drugs, such as cisplatin (cDDP), carboplatin and oxaliplatin, which are often associated to severe toxicity and acquired resistance [1,2,3]. For instance, the complex of palladium with spermine (Pd_2_Spm) has exhibited promising antiproliferative, antimigratory and antiangiogenic properties towards the human triple-negative breast cancer (TNBC) MDA-MB-231 cell line [4,5,6,7], among further beneficial properties in other types of cancer cell lines (e.g., leukemia [8], osteosarcoma [9,10], and squamous [11] and ovarian carcinomas [12]). In addition, Pd_2_Spm has been recently reported to induce a positive response in a cell-derived xenograft (CDX) mouse model of TNBC, evidenced by the extension of reduction in tumor growth and proliferation, comparable to those achieved by cDDP treatment [7]. In addition, exposure of healthy BALB/c mice to Pd_2_Spm to investigate drug induced toxicity effects has unveiled a favorable pharmacokinetic profile (including biodistribution), as compared to cDDP [13], properties which should translate into tumor-bearing mice.

The full understanding of drug efficiency and toxicity mechanisms may benefit from an improved knowledge of the metabolic response of tumors and overall organism (organ-localized or systemic) to treatment, and untargeted metabolomics is a valuable tool in this respect. In fact, metabolomic studies of the above mentioned CDX mouse model of TNBC showed that tumor response to Pd_2_Spm was accompanied by a more pronounced and distinct metabolic response, compared to cDDP (specifically comprising deviations in energy, membrane, nucleotides and one-carbon metabolisms) [14], which suggested that different drug mechanisms may be active. However, the concomitant metabolic response of different CDX mice organs to each of the two drugs has not, to our knowledge, been investigated up to this date, while it may provide meaningful information on drug toxicity/secondary effects. In healthy BALB/c mice, a faster post drug exposure recovery of the original homeostatic metabolic signatures of kidney, liver, breast tissue and brain has been noted for Pd_2_Spm, compared to cDDP, this efficient metabolic recovery suggesting a lower toxicity of the Pd(II) agent [15,16]. In this animal model, the decreasing magnitude of metabolic changes in kidney to liver and then to mammary gland exposed to either Pd_2_Spm or cDDP [15] seemed to reflect a decreasing accumulation of Pt(II) or Pd(II) in these organs during the steady phase (ca. 50–100 ng of metal/g of tissue/μg of metal dose administered in kidney, ca. 20 for liver and 3–5 for mammary gland). Interestingly, in spite of only negligible amounts (<1 ng/g/μg) of these metals reaching the brain of healthy BALB/c mice, when normalized to administered dose [13], both cDDP and Pd_2_Spm were found to significantly affect brain metabolism, although in distinct ways, as previously reported [16]. Compared to cDDP, Pd_2_Spm induced an earlier (1 h) and stronger effect on brain metabolome, particularly on purine metabolism (AMP and ADP pools), which was, however, quickly recovered as were the levels of other metabolites. This suggested an overall more effective protective response of brain metabolism towards oxidative stress, as well as towards alterations in amino acid metabolism and membrane remodeling. These results on healthy BALB/c mice emphasized the importance of indirect drug impacts on brain metabolism, particularly in the case of compounds which do not cross the blood-brain-barrier (BBB) as seems to be the case for cDDP and Pd_2_Spm. Indeed, a major limitation of chemotherapeutic treatments with high and cumulative doses of platinum(II)-drugs is neurotoxicity, leading to acute neuropathies, especially in the peripheral nervous system [17,18,19,20], and persistent cognitive impairment at a late- or post-stage of therapy (known as chemobrain) [21,22,23]. Other metabolomic studies of cDDP on mice brain have characterized brain (and liver) extracts from Sprague-Dawley (SD) rats [24] and hypothalamus lipidic extracts (in connection with plasma Nuclear Magnetic Resonance (NMR) metabolic characterization) of Lister hooded rats, to investigate the possible protective role of the natural compound cannabigerol [25]. As brain metabolome characteristics are not expected to be comparable between different mice models, or between healthy and tumor-bearing states, it is of relevance to characterize the brain metabolic profile in tumor-bearing CDX mice as a response to either cDDP or Pd_2_Spm, for which low or no metal amounts are expected to accumulate in brain, judging by the results obtained for BALB/c mice.

This paper presents, for the first time to our knowledge, an assessment of the metabolic response of mice’s brain and liver to the administration of Pd_2_Spm or cDDP (as a drug reference) in a CDX mice model of TNBC, using metabolomics. For both organs, polar and lipophilic extracts were obtained and analyzed by NMR. Metabolic deviations detected in brain tissue were further studied through their correlation to those found in liver, in order to account for brain-liver axis interplay, expected to reflect communication between brain and the organism periphery through neurotransmitter-mediated hormonal regulation and subsequent regulation of systemic metabolism by liver. Putative biochemical interpretations of brain and liver metabolic responses to either Pd_2_Spm or cDDP are advanced, contributing to an improved understanding of the drugs’ effects on the brain of exposed subjects.

## 2. Results

### 2.1. NMR Spectra of CDX Mice Brain and Liver Extracts

**Figure 1:**
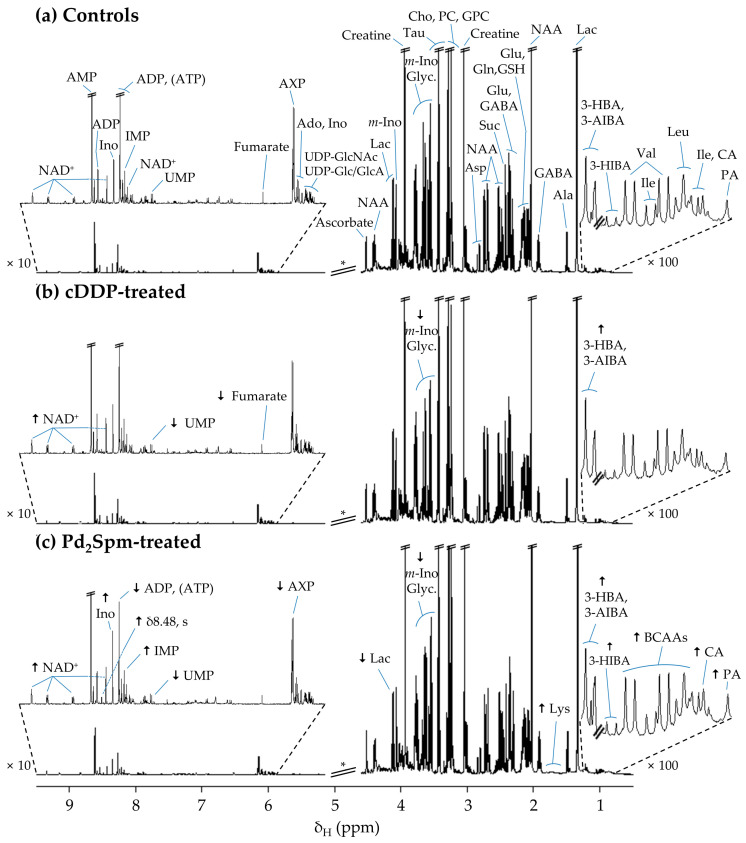
Average 500 MHz ^1^H Nuclear Magnetic Resonance (NMR) spectra of an aqueous profile of brain from a cell-derived xenograft (CDX) mouse model of TNBC exposed to (**a**) vehicle solution (phosphate-buffer saline, PBS), (**b**) cDDP (2 mg/kg/day), and (**c**) Pd_2_Spm (5 mg/kg/day). * Cut-off of water suppression region (δ 4.6–5.1 ppm), not considered in the multivariate analysis. The arrows identify metabolic variations found with visual inspection of spectra from each treated group compared to controls (↑, increase; ↓, decrease). Abbreviations: 3-letter code for amino acids; 3-AIBA, 3-aminoisobutyrate; 3-HBA, 3-hydroxybutyrate; 3-HIBA, 3-hydroxyisobutyrate; Ado, adenosine; ADP, adenosine diphosphate; AMP, adenosine monophosphate; ATP, adenosine triphosphate; AXP, adenosine nucleotides, AMP, ADP and ATP; BCAAs, branched-chain amino acids (ile, leu and val); CA, cholate; Cho, choline; GABA, γ-aminobutyrate; Glyc., glycerol; GPC, glycerophosphocholine; GSH, glutathione (reduced); IMP, inosine mono-phosphate; Ino, inosine; *m*-Ino, myo-Inositol; Lac, lactate; NAA, *N*-acetyl-aspartate; NAD^+^/NADH, nicotinamide adenine dinucleotide (oxidized/reduced); NADPH nicotinamide adenine dinucleotide phosphate (reduced); PA, pantothenate; PC, phosphocholine; Suc, succinate; Tau, taurine; UDP-Glc/GlcA, UDP-Glucose/Glucuronate; UDP-GlcNAc, uridine diphosphate *N*-acetylglucosamine; UMP, uridine monophosphate.

The average ^1^H NMR spectra of the aqueous extracts of untreated CDX mice brain (Figure 1a) reflect the complex composition of the sample, rich in amino acids, organic acids, choline compounds and several nucleotides and derivatives, in agreement with previous metabolomic studies on healthy animals, namely BALB/c mice [16] and SD rats [26,27]. It is important to note that the metabolic features of controls (untreated xenografts) should, naturally, not be misinterpreted as descriptive of a healthy state as they may bear effects of tumor growth on the organism. Apparent visual spectral changes are observed with cDDP treatment (Figure 1b) compared to controls (untreated mice) (Figure 1a), comprising increased levels of 3-hydroxybutyrate (3-HBA), 3-aminoisobutyrate (3-AIBA) and oxidized nicotinamide adenine dinucleotide (NAD^+^), along with decreased levels of *myo*-inositol, glycerol, fumarate and uridine monophosphate (UMP). Most of these changes were also observed for Pd_2_Spm-treated samples (Figure 1c), although several additional changes were also noted, namely increased levels of pantothenate (PA), cholate (CA), 3-hidroxyisobutyrate (3-HIBA), branched-chain amino acids (BCAAs: Ile, Leu, Val), Lys, inosine monophosphate (IMP), inosine (Ino), and an unassigned singlet at δ 8.48 ppm. Furthermore, decreased levels were observed for lactate, and mono-, di-, triphosphate adenine nucleotides (AXP: AMP, ADP, ATP). As the above observations are based on average spectra, it is important to seek their confirmation by statistical analysis (Section 2.2), although it already seems clear that Pd_2_Spm induces a stronger impact on the polar metabolome of brain, as compared to that exerted by cDDP.

The average ^1^H NMR spectrum of the lipophilic extracts of untreated brain (Figure S1a) is characterized by a predominance of cholesterol (mostly in its free form), saturated and unsaturated fatty acids (FA), as well as several classes of phospholipids (PL), in broad agreement with previous reports [16,26]. In comparison to untreated samples, treatment with either metal agent did not induce clear visual changes in the average spectra of lipophilic extracts.

Regarding liver aqueous extracts (Figure S2), the metabolic profile obtained for control (untreated) samples (Figure S1a) is similar to that reported for healthy rodents, such as BALB/c [16,28], C57BL6 mice [29] or SD rats [30]. Such profile includes relatively high contents of lactate, choline compounds, betaine, taurine and glucose, and lower contents of adenine nucleotides and other nitrogenous bases derivatives. In liver, cDDP seemingly leads to visually increased levels of Gln, reduced glutathione (GSH), Tyr, His, AXP nucleotides and oxidized nicotinamide adenine dinucleotide phosphate (NADP^+^), along with decreases in malonate and unassigned doublet at δ 4.03 ppm. On the other hand, the Pd(II) agent seems to also induce decreases in malonate and unassigned δ 4.03 ppm doublet, as well as in AXP nucleotides (particularly AMP), UMP and pseudouridine.

In addition, the average ^1^H NMR spectra of lipid extracts of control liver (Figure S1b) is also broadly consistent with previous reports on healthy rodents [15,29], exhibiting the presence of peaks from cholesterol (combination of free and esterified forms), PL (mostly phosphatidylcholine (PTC), phosphatidylethanolamine (PTE) and sphingomyelins (SM)) and triacylglycerols (TG), as well as from FA with different saturation degrees. As observed for the brain, no clear visual changes could be observed in the average spectra of liver lipophilic extracts, upon either drug treatment.

### 2.2. Effect of cDDP and Pd_2_Spm on CDX Mice Brain Profiling

**Figure 2:**
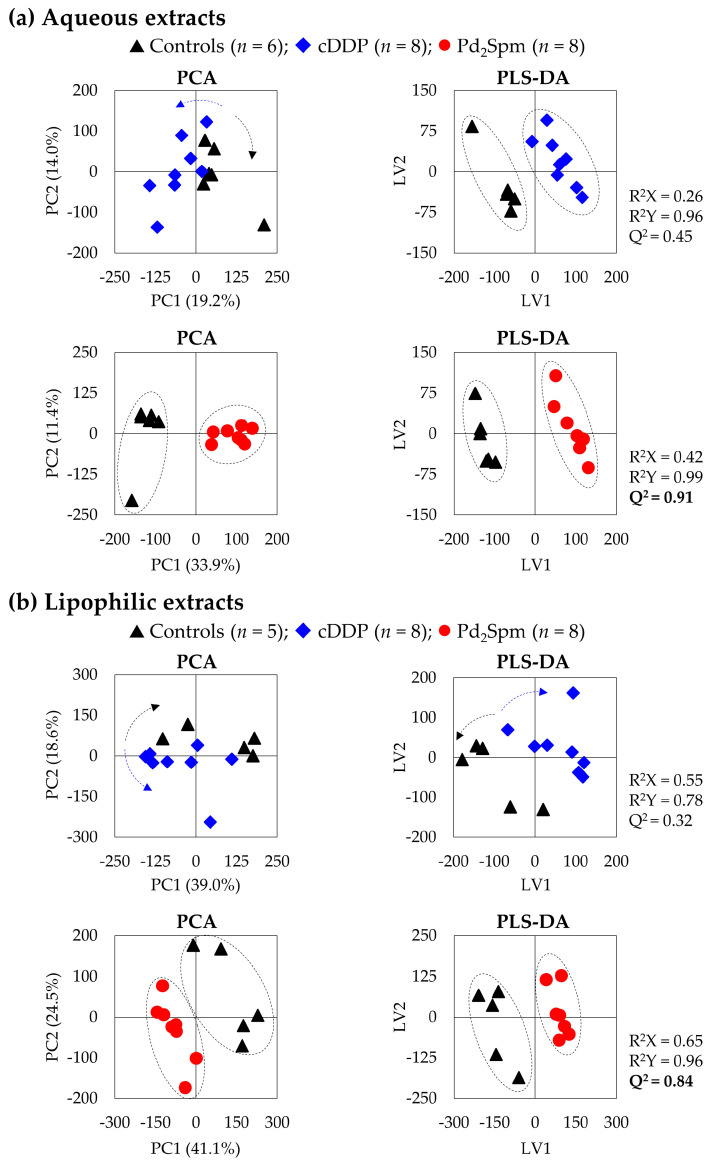
Score scatter plots of PCA (right) and PLS-DA (left) models for ^1^H NMR spectra of (**a**) aqueous and (**b**) lipophilic extracts of brain from CDX mice of TNBC, considering the pairwise analysis of the treated groups, with either cDDP or Pd_2_Spm, compared to controls (controls, black triangles, *n* = 6 or 5 for aqueous or lipophilic extracts, respectively; cDDP-treated, blue diamonds, *n* = 8; Pd_2_Spm-treated, red circles, *n* = 8). Validation parameters (R^2^ and Q^2^) are indicated for each PLS-DA model, with Q^2^ values > 0.5 highlighted in bold, indicating high predictive power and, thus, robust classes separation.

The group separation observed through principal component analysis (PCA) of the spectra of brain aqueous extracts (Figure 2a, left) reflects the significant impacts of both drugs on brain metabolic profile, with a more pronounced effect for Pd_2_Spm (34% of variability explained by PC1, compared to 19% for cDDP-treated mice vs. controls). Group separation is visible in partial least-squares discriminant analysis (PLS-DA) scores for both drugs, and the higher predictive power (or robustness) of Q^2^ (0.91) for Pd_2_Spm-treated vs. controls (compared to Q^2^ 0.45 for cDDP-treated vs. controls) confirms the stronger impact of the palladium agent, already suggested by a simple visual inspection of the spectra.

Regarding the lipid extracts (Figure 2b), weaker PLS-DA models are observed (in agreement with no clear visual changes in the lipidic spectra, upon drug treatment), but some group separation is noted for Pd_2_Spm-treated samples, as indicated by PLS-DA Q^2^ 0.84 for Pd_2_Spm-treated vs. controls (compared to Q^2^ 0.32 for cDDP vs. controls).

**Figure 3:**
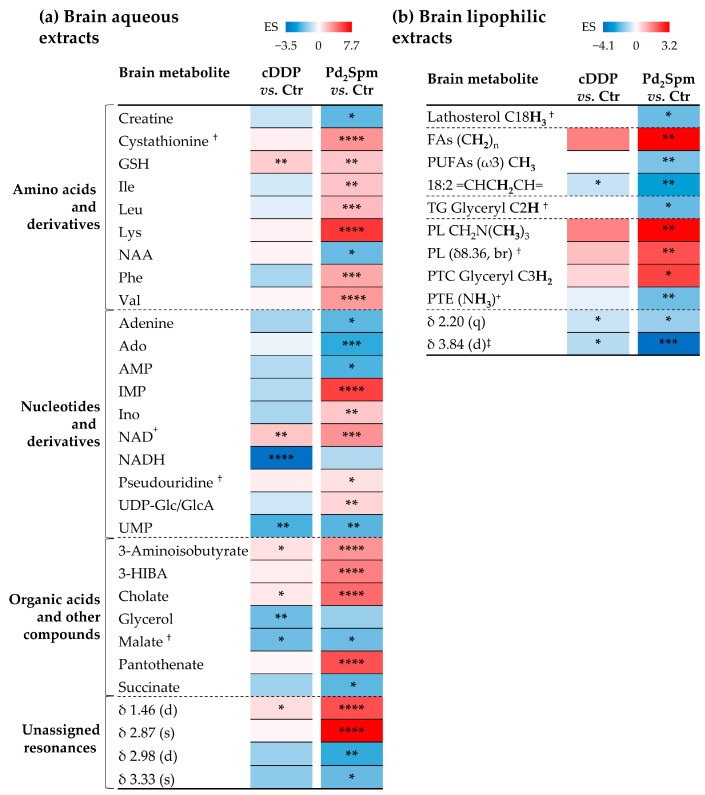
Heatmaps illustration of the effect size (ES) values of statistically significant variations in the (**a**) aqueous, and (**b**) lipophilic extracts of the brain of TNBC xenografts exposed to cDDP or Pd_2_Spm in comparison to controls. Metabolic variations are colored from blue to red, representing an increasing ES scale from negative to positive values, respectively. Abbreviations: (**a**) as defined in Figure 1; (**b**) FAs, fatty acids; PLs, phospholipids; PTC, phosphatidylcholine; PTE, phosphatidylethanolamine; PUFAs, polyunsaturated fatty acids; TG, triacylglycerols; s, singlet; d, doublet; q, quartet; br, broad signal. ^†^ Tentative assignment. ^‡^ Partial integration of resonance peak. * *p*-value < 5.0 × 10^−2^; ** *p*-value < 1.0 × 10^−2^; *** *p*-value < 1.0 × 10^−3^; **** *p*-value < 1.0 × 10^−4^.

Peak integration and effect size (ES) calculation (Table S1 and Figure 3) confirm a stronger effect of Pd_2_Spm on amino acids and derivatives, compared to the effect exhibited by cDDP (namely, marked increases in the three BCAA, Lys (the highest increase, Table S1), Phe, cystathionine and GSH (a common feature to both drug-treated groups), whereas decreases are noted in creatine (Cr) and *N*-acetylaspartate (NAA, an aspartate-derived neurotransmitter). Pd_2_Spm also induces strong changes in the brain nucleotides and derivatives (Table S1 and Figure 3), namely significant increases in IMP, Ino, NAD^+^, UDP-Glucose/Glucuronate (UDP-Glc/GlcA) and pseudouridine (a C-glycosyl isomer of uridine found in RNA), and decreased levels of adenine, adenosine (Ado), AMP and UMP (the latter in common with cDDP-treated mice). In comparison, cDDP induces only slight variations in the polar metabolome of mice brain (Figure 3a), although a significant NADH decrease is observed, contrary to the Pd_2_Spm, which does not significantly impact on NADH levels. In spite of the low signal-to-noise ratio of NAD^+^ and NADH peaks, differences between controls and each of the drug-treated groups are visually clear (Figure S3a) and translate into significant ES values (Table S1) and different NAD^+^/NADH ratios (Figure S3b): 4.6 ± 0.88 for controls, 9.2 ± 1.6 for cDDP, and 7.3 ± 1.6 for Pd_2_Spm. Since NAD^+^/NADH is a common indicator of intracellular redox state regulation [31], these results suggest a more effective response of the brain to oxidative stress in the case of Pd_2_Spm treatment. The metabolic response of the brain to this Pd(II) complex is complemented with marked increases in the acids 3-AIBA, 3-HIBA, CA and PA, the latter left unchanged by cDDP. In addition, both drugs induce a decrease or weak decreasing tendencies in glycerol, malate and succinate. Four still unassigned peaks are notably altered in the Pd_2_Spm-treated group, reinforcing the higher impact of this complex on the brain metabolism, as compared to cDDP.

Although the lipophilic metabolome of the brain is much less affected than the polar metabolome, a qualitatively similar profile of chemical changes is noted for both drugs (Figure 3b), although more significant (higher magnitude and statistical relevance) for Pd_2_Spm (Figure 3b and Table S2, top). Such variations comprise a rise in PTC and FA (probably as part of PL), and a decrease in PTE, TG, ω3 polyunsaturated FAs (PUFAs), linoleic acid (18:2 Δ^9,12^) and lathosterol, apart from an unassigned resonance at δ 3.84 ppm. Further considerations on average FA chain length and unsaturation degree could not be advanced, due to the strong overlap of cholesterol peaks (C26H_3_ and C27H_3_ resonances) with terminal FA methyl resonances, hindering their use as reliable indicators of the total FA amount.

### 2.3. Effect of cDDP and Pd_2_Spm on CDX Mice Liver Profiling

**Figure 4:**
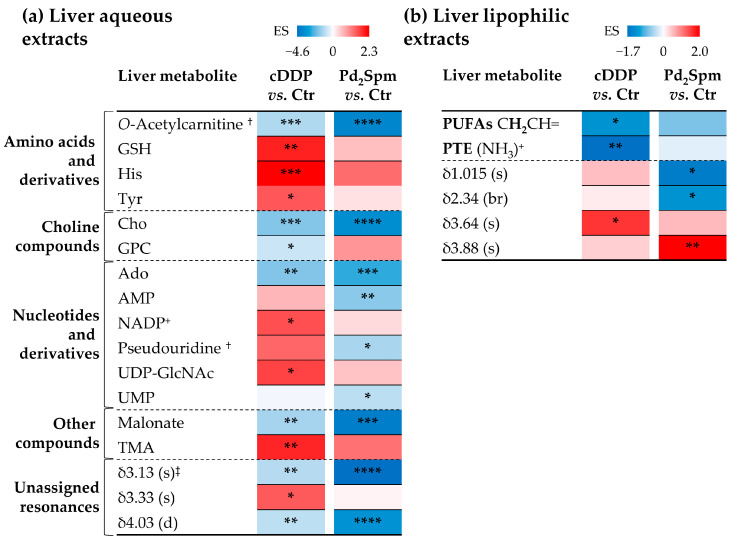
Heatmaps colored according to the effect size (ES) of variations in the (**a**) aqueous, and (**b**) lipophilic extracts of liver of TNBC mice. Abbreviations: NADP^+^ nicotinamide adenine dinucleotide phosphate (oxidized); TMA, trimethylamine; other abbreviations as defined in Figure 1; s, singlet; d, doublet; br, broad signal. ^†^ Tentative assignment. ^‡^ Partial integration of resonance peak. * *p*-value < 5.0 × 10^−2^; ** *p*-value < 1.0 × 10^−2^; *** *p*-value < 1.0 × 10^−3^; **** *p*-value < 1.0 × 10^−4^.

Contrary to the results obtained for the brain, PCA of the spectral data obtained for liver aqueous and lipophilic extracts (Figure S4a,b, respectively) does not reveal any group separation, which indicates that no significant changes have been induced by the drug in this organ. However, in the case of aqueous extracts (Figure S4a), PLS-DA provides satisfactory models (Q^2^ 0.70 and 0.59 for cDDP and Pd_2_Spm, respectively), with a tendency for cDDP to impact more strongly on liver polar metabolome than Pd_2_Spm. A similar tendency is noted through PCA and PLS-DA obtained for lipophilic extracts (Figure S4b). The varying polar metabolites (Figure 4) reveal similar signatures for both drugs, although with enhanced statistical relevance in cDDP-treated mice, namely an increase in GSH, His, Tyr, NADP^+^, UDP-*N*-acetylglucosamine (UDP-GlcNAc), trimethylamine (TMA) and unassigned peak at δ 3.33 ppm, and a decrease in glycerophosphocholine (GPC). On the other hand, the liver of Pd_2_Spm-treated animals is more depleted in *O*-acetylcarnitine (ALC), choline (Cho), Ado, AMP, pseudouridine, UMP, malonate and unassigned resonances at δ 3.13 and 4.03 ppm.

Regarding liver lipophilic extracts, a more enhanced decrease in PUFAs and PTE is observed with cDDP treatment, whereas only weak decreasing tendencies are seen for Pd_2_Spm (Figure 4 and Table S2). The average FA chain length and unsaturation degree were found not to change significantly between the untreated samples and any of the two treated groups. In addition, the pattern of changes in a few unassigned resonances (Figure 4) is different between drugs but difficult to interpret without further assignment.

### 2.4. Intra- and Inter-Organ Correlations for Brain and Liver Polar Profiles

**Figure 5:**
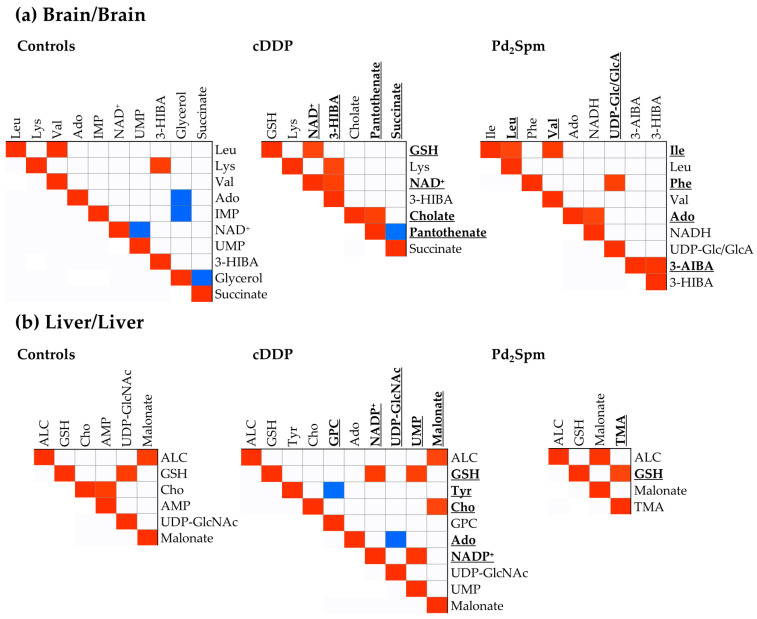
Spearman correlations between the significant metabolic variations within each tissue, namely (**a**) brain and (**b**) liver, for controls (left), the cDDP-treated group (middle), and the Pd_2_Spm-treated group (right), considering an absolute correlation coefficient of 0.90. All *p*-values of correlations are comprehended between 0.05 and 0.001 and are then considered statistically relevant. Bold and underlined metabolites represent new correlations regarding those found in controls (left). Abbreviations: ALC, *O*-acetylcarnitine. Other abbreviations as defined in Figure 1 and Figure 4.

**Figure 6:**
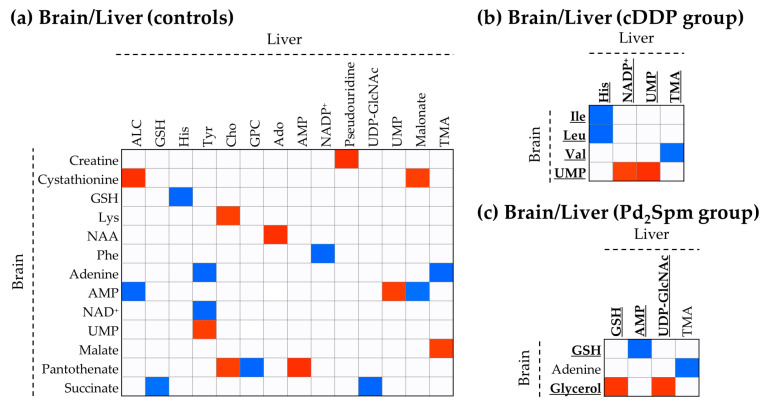
Spearman correlations between significant variations between liver and brain of (**a**) controls and groups treated with (**b**) cDDP or (**c**) Pd_2_Spm, regarding an absolute correlation coefficient of 0.80. The correlations are statistically significant, exhibiting *p*-values between 0.05 and 0.01. Bold and underline metabolites represent new correlations regarding those found in controls. Abbreviations as defined in Figure 1, Figure 4 and Figure 5.

Interpretation of the above observations is challenging and necessarily requires additional research. Metabolite correlation maps may be useful to single out the main fates of particular metabolites. In these maps, strong positive or negative correlation suggest that the metabolites involved are biochemically correlated and following related dynamics, as opposed to metabolites involved in multiple pathways/dynamics for which no clear correlations have become visible. Correlation studies descriptive of untreated mice, and cDDP- and Pd_2_Spm-treated groups were obtained for the brain and liver alone (Figure 5) as well as between the brain and liver (Figure 6). In the absence of treatment, the brain metabolism is expressed by six strong correlations (Figure 5a, left): two positive ones involving amino acids (Val/Leu (+), Lys/3-HIBA (+)); and negative correlations of glycerol with Ado, IMP and succinate, and of UMP with NAD^+^. The correlation pattern induced by cDDP treatment (Figure 6a, center) maintains the Lys/3-HIBA (+) correlation, adding NAD^+^/GSH (+) and NAD^+^/3-HIBA (+), as well as an interesting interplay between PA with succinate (−) and CA (+). Treatment with Pd_2_Spm maintains correlations within some BCAA, as seen in untreated mice (namely: Ile with Leu (+) and Val (+)) and adds 3-HIBA/3-AIBA (+), UDP-Glc/GlcA/Phe (+) and NADH/Ado (+) correlations (Figure 5a, right). Hence, the brain metabolite patterns differ significantly upon treatment, mainly involving the metabolism of amino acids (specifically, BCAA, Lys and Phe), 3-HIBA, GSH and NAD^+^/NADH. In addition, correlations for PA/CA/succinate and UDP-Glc/GlcA appear to be specifically observed for cDDP- and Pd_2_Spm-treated animals, respectively.

Regarding liver (Figure 5b), it is interesting to note that the malonate/ALC (+) correlation is observed in all three groups, suggesting a consistent underlying regulation between both FAs synthesis and β-oxidation, respectively. Qualitatively, controls and Pd_2_Spm correlation maps are the simplest (with three and two positive correlations alone, respectively), with GSH correlating to UDP-GlcNAc (+) in controls and to TMA (+) in the Pd_2_Spm-treated group. In addition, controls exhibit an AMP/Cho (+) correlation. The correlation map is more complex for the cDDP-treated group (Figure 5b, center), with malonate also correlating with Cho (+) (besides ALC), and a distinct pattern involving GSH, with correlations to UMP (+) and NADP^+^ (+). Moreover, two new negative correlations are noted: GPC/Tyr (−) and UDP-GlcNAc/Ado (−).

When the two organ matrixes are correlated (Figure 6), the correlation pattern of untreated animals is strikingly complex in terms of the number of metabolites involved, whereas the inter-organ patterns for treated animals are both remarkably simple. Notably, cDDP treatment highlights a negative correlation of the brain BCAA (which does not vary significantly for cDDP) with liver His (which increases in cDDP-treated liver) and with TMA (which also increases in cDDP-treated liver). This means that increased liver His and TMA levels may relate to lower BCAA in the brain. In addition, the brain UMP (decreased upon treatment with both drugs) correlates positively with liver UMP (only slightly decreasing with cDDP) and NADP^+^ (increased by cDDP treatment). This indicates that, in the case of cDDP, liver and the brain UMP directly influences each other, with NADP^+^ involved in liver UMP metabolism. The same clear relationship is not observed in Pd_2_Spm administration. The simpler liver pattern for Pd_2_Spm-treated animals comprises GSH/glycerol (+) and GSH/AMP (−), in addition to glycerol/UDP-GlcNAc (+) and adenine/TMA (−), the latter being present in untreated samples.

## 3. Discussion

### 3.1. Metabolic Impact of Drugs on the Brain

Bearing in mind that negligible amounts Pd(II) or of Pt(II) (<1 ng of metal/g of tissue/μg of metal dose administrated) have been found in the brain of healthy BALB/c mice, compared to other organs [13], reflecting an effective protective role of the BBB towards these agents, it was interesting to observe that Pd_2_Spm exerted a particularly strong impact on the brain metabolism (particularly evidenced in the polar metabolome). The possibility that the responsive metabolites could be reflecting an indirect neuroinflammation or neuronal damage was firstly considered. Neuroinflammation is mediated by the progressive activation of endothelial cells and of central nervous system (CNS) glia cells (astrocytes and microglia) [32], the latter having often been associated with increased levels of *myo*-inositol, choline-containing compounds (Cho, phosphocholine (PC) and GPC) and total Cr (tCr, comprising Cr, and phosphocreatine, PCr) [33]. Indeed, *myo*-inositol is an important indicator of glia cells behavior, due to its glial osmolyte function and its higher concentration in such cells, compared to neurons. Higher levels of Cho compounds have been related to higher cellular turnover, and higher tCr has been interpreted as reflecting energy metabolism regulation [33]. In this work, none of the two drugs perturb the levels of *myo*-inositol and Cho compounds significantly, qualitative decreasing tendencies having been noted (namely for *myo*-inositol, Cho and GPC), along with a relevant decrease in Cr upon Pd_2_Spm exposure (similar tendency in the cDDP-treated group). This Cr decrease for both drugs may reflect a lower creatine kinase activity (BCK isoform [34]) and a subsequent reduction in ATP production through conversion of PCr to Cr. Hence, the expected metabolic profile characteristic of neuroinflammation does not seem to be corroborated by our results. The apparent tendency for depletion in cerebral osmolytes (based on the *myo*-inositol decreasing tendency) is also consistent with decreases in glycerol (also recognized as an osmolyte [35]), although the variation is more pronounced for cDDP treatment.

Furthermore, the loss of neuronal density or function, associated with neuronal integrity injury, has been usually associated with diminished levels of neurometabolites, such as Glu, NAA and *N*-acetylaspartylglutamate [33]. In particular, neuronal demyelination is known to be accompanied by a decrease in NAA, along with increases in glial markers (*myo*-inositol, choline-compounds, and tCr), lipids and lactate, reflective of cell membrane degradation and a metabolic switch to anaerobic respiration, respectively [33,36]. Our results show that none of the above-mentioned changes occur either with cDDP or Pd_2_Spm treatment, except for the decreased levels of NAA in the Pd_2_Spm-exposed group (although with relatively low statistical relevance (ES −1.4 ± 1.2, *p*-value 0.048) compared to most variations observed). This suggests that the overall integrity and function of the brain seem to be largely unaffected by either drug. For Pd_2_Spm, this hypothesis is further corroborated by the qualitative tendencies of increased levels of Glu and γ-aminobutyric acid (GABA), the two major excitatory and inhibitory neurotransmitters of CNS, respectively [37]. Thus, neither cDDP nor Pd_2_Spm exposure seems to induce neuronal damage or inflammation, at least considering the reported metabolic indicators.

Regarding the observed changes in the brain polar metabolome upon Pd_2_Spm treatment, important increases were noted in many amino acids and derivatives, apart from Cr and NAA, whereas only weak tendencies were found in the cDDP-treated group. One of the main observations regards the marked increase in all three essential BCAA. These amino acids play an immensely important role in rodent and human brains [37] as nitrogen donors for proteins and/or neurotransmitters synthesis, namely, to form Glu, GABA, as well as Gln. The latter mediates Glu accumulation in neurons, through the Glu/Gln cycle, and helps to prevent neuron excitotoxicity due to excessive extracellular Glu levels [37]. No significant changes were noted in either Glu, Gln or GABA for any of the drug-treated groups, which indicates that the levels of these amino acids are successfully regulated in all samples. However, in the case of Pd_2_Spm, this seems to be ensured by larger pools of the brain BCAA (no corresponding BCAA changes having been seen in the liver, so that the exact origin of their increase in the brain remains unclear). The increased levels of Phe (and a similar qualitative tendency for Tyr) may relate to serotonin and catecholamine synthesis [38], corroborating the hypothesis of a higher demand for neurotransmitter synthesis upon treatment with the Pd(II)-complex. It is possible, however, that the increase in amino acids may also arise, at least in part, from intracellular protein degradation, and this would be an issue for further investigation. In the Pd_2_Spm group, important increased levels of Lys (also an essential amino acid), cystathionine and GSH were seen. Lys may also be a precursor of Glu in mammalian CNS [39], probably through the pipecolate pathway, proposed to preferentially take place in the brain, rather than the alternative saccharopine pathway, which has been suggested to predominate in extracerebral tissues [40]. The higher Lys levels in the case of Pd_2_Spm treatment may arise for the same reason as elevated BCAA, namely, to ensure that the Glu pool is maintained suitably regulated. Regarding cystathionine and GSH, it is interesting to note that both are elevated in Pd_2_Spm-treated mice, whereas only GSH is increased in cDDP-treated animals (to a similar extent as in the Pd_2_Spm group). Therefore, GSH seems to be required in both treatments for oxidative stress protection of the brain [41], but in the presence of Pd_2_Spm, an elevated pool of cystathionine (an intermediate in the synthesis of Cys and GSH, by glutamate-cysteine ligase, GCL, and glutathione synthetase, GSS), also seems to be required. This appears to indicate that in the brain of Pd_2_Spm-treated animals, cystathionine is called upon to reinforce GSH biosynthesis for a more effective antioxidant protection, probably also enhanced, in part, due to the presence of the spermine polyamine (a known biogenic antioxidant). Indeed, a higher antioxidant protection is consistent with the lower NAD^+^/NADH ratio (7.3 ± 1.6) found for the Pd_2_Spm group, compared to cDDP-exposed animals (9.2 ± 1.6). Notably, NAD^+^ levels are elevated in both treated groups relatively to controls (Table S1), but more so in the Pd_2_Spm samples, for which NADH levels remain equivalent to those in the controls. This suggests that increased NAD^+^ levels do not arise directly from NADH oxidation (e.g., through glycolysis and the tricarboxylic acid cycle, TCA) but, rather, from an alternative source. In turn, the strong NADH depletion in the cDDP group is consistent with a prompt requirement for an antioxidant response. Still regarding NAD^+^ levels, these are usually higher in cancer compared to normal cells, due to an upregulation of NAD^+^ synthesis (mainly through the NAD^+^ salvage pathway) [42], and therapy may have a decreasing effect on NAD^+^. However, no significant differences were observed between untreated tumors and those treated with either cDDP or Pd_2_Spm, although a slight tendency for higher NAD^+^ levels has been observed for Pd_2_Spm-treated tumors (only when directly compared to cDDP-treated tumors and to a low extent ES 1.35 ± 1.21, *p*-value 0.039) [14]. It is therefore possible that a small extra amount of NAD^+^ is transported from the tumors through the BBB (as previously reported [43]) in Pd_2_Spm-treated animals. However, other experimental evidence has indicated that NAD^+^ levels may increase due this transporters’ role in mediating synaptic plasticity and neuronal stress resistance [44]. If this is the case, it would suggest that a NAD^+^ elevation (particularly enhanced in the Pd_2_Spm group, almost up to 2-fold) may indicate a favorable adaptive response of the brain. Such a response has been demonstrated in the context of Alzheimer’s’ disease (AD) [45], where NAD^+^ supplementation in mouse models resulted in the counteraction of inflammatory processes, by reducing activated glia and pro-inflammatory cytokines.

Still regarding nucleotides and their derivatives, the Pd_2_Spm signature distinctly exhibits a shift in purines metabolism, possibly directed at raising Ino and IMP levels at the expense of other purines, such as adenine, Ado and AMP. This may indicate a positive adaptation of cells towards a neuroprotective mode, since Ino exhibits anti-inflammatory and antioxidant properties [46], as revealed in the treatment of several neurodegenerative diseases [46,47]. The depletion of UMP, observed to comparable extents in both treated groups, may be due to this metabolite maintaining neuronal cell membranes architecture, through regulation of choline metabolism and production of phosphatydilcholine (PTC) [48,49]. However, a PTC increase was only significant for the Pd_2_Spm-treated group (showing an increasing tendency with cDDP), thus suggesting a corresponding higher extension of the role of UMP in PTC production. In addition, other aspects of uridine metabolism are indeed enhanced in the Pd_2_Spm group, such as the rise in UDP-Glc/GlcA (contrary to cDDP), probably associated with shifts in glucosaminoglycans and proteoglycan metabolism [50], and in pseudouridine (Ψ, tentative assignment). Elevated pseudouridine has also been reported in the urine of patients suffering from mild- to-moderate AD [51], revealing an implication of this compound in neuronal functions, possibly as a repercussion of oxidative stress. The above observations indicate uridine metabolism as an interesting target of future enzymatic studies, in order to demonstrate or discard the above putative hypotheses.

The strong Pd_2_Spm-induced increases in brains’ 3-AIBA, 3-HIBA, PA and the unconjugated bile acid CA, occur in tandem with decreases in malate and succinate for both cDDP and Pd_2_Spm-treated samples (succinate being more enhanced in the presence of Pd_2_Spm, probably reflecting a defective adaptation within the TCA cycle activity). 3-HIBA and 3-AIBA are intermediates of both Val and thymine catabolism, their increase possibly evidencing some extent of impairment in the activity of methylmalonate semialdehyde dehydrogenase [52], which would result in lower levels of succinyl-CoA (and hence of succinate) in the TCA cycle. This effect is only hinted in cDDP-treated animals. PA is a precursor of endogenous cholesterol synthesis in the liver [53], the two compounds being closely related to isomers. It is possible, therefore, that the marked Pd_2_Spm triggered PA increase in brain may relate to a lathosterol decrease, reflecting drug-specific adaptations affecting cholesterol synthesis. In addition, the bile acid salt CA (which may cross the BBB [54]) may also be involved in hormonal signaling via cholesterol catabolism in the liver-gut-brain axis [54,55], possibly adding to the effect expressed by lathosterol depletion. The remaining Pd_2_Spm-induced changes in brain lipophilic compounds include stronger depletion in TG, unsaturated FA and PTE and an increase in PTC (as mentioned earlier in relation to UMP and choline metabolisms). These suggest more enhanced alterations in cell membrane properties, such as fluidity and curvature [56] in the case of Pd_2_Spm treatment, an issue to be investigated in future studies.

Brain intra-correlations for untreated mice show the importance of BCAA metabolism, including a relationship between Lys and 3-HIBA which may also indirectly reflect BCAA metabolism, since 3-HIBA is related to Val degradation, as formerly reported [52]. Metabolic relationships are also evidenced between glycerol and either nucleosides/nucleotides (namely adenosine and IMP) or succinate. The UMP/NAD^+^ relationship may reflect the role of NAD^+^ as electron receptor for the reaction catalyzed by carbamoyl phosphate synthetase II, to produce orotate, a precursor of UMP, within the de novo pyrimidines biosynthesis in the brain [57]. It is important to note analysis of the correlation maps, without further supporting experimental biochemical data, may lead to overinterpretation, so that the discussion below will consider only the main variations observed upon drug treatment.

Exposure to cDDP leads to the loss of a clear BCAA pattern, which suggests multiple fates for BCAA, probably currently also used as anaplerotic sources for the TCA cycle (Leu is ketogenic, Val is gluconeogenic and Ile is both ketogenic and gluconeogenic [37]), which may induce different rates of usage for each amino acid, therefore explaining the absence of clear correlations. Conversely, the positive correlation between Lys and 3-HIBA is maintained, although the latter is also connected to NAD^+^ and GSH, suggesting a different interplay related to oxidative protection mechanisms possibly due to the higher oxidative stress observed in the brain of animals in this group. Furthermore, in cDDP treatment, succinate seems related to cholesterol metabolism (as shown by its correlations to PA and CA), in spite of succinate and PA only varying upon Pd_2_Spm treatment and CA showing an almost 4-fold increase in the same group. Hence, the correlation maps usefully show that PA and CA relationships to cholesterol metabolism are also present in the cDDP group, in spite of not translating into meaningful variations of those metabolites. We also suggest that the PA/succinate (−) correlation is probably due to PA giving rise to CoA [58,59], which then enters the TCA, activating it to some extent and thus using up succinate (although no significant decrease in this acid was seen for the cDDP group).

Exposure to Pd_2_Spm maintains a BCAA pattern (although slightly different from that observed in the absence of treatment), which may be indicative of a suitable regulation of the Glu pool. On the other hand, the positive correlation between Lys and 3-HIBA is replaced by a 3-HIBA/3-AIBA (+) correlation, consistent with an enhancement of their interconversion pathway related to the above-mentioned possible impairment of methylmalonate semialdehyde dehydrogenase activity [52], and a subsequent marked decrease in succinate. Interestingly, Lys increases significantly upon Pd_2_Spm treatment (ES 6.0 ± 2.5, *p* < 5.9 × 10^−8^) upon Pd_2_Spm treatment, this effect not being observed upon cDDP-exposure. The fact that the Lys/3-HIBA (+) correlation is lost in the former group suggests that Lys is probably also engaged in other metabolic pathways. The Pd_2_Spm-treated samples are uniquely characterized by a positive relationship between UDP-Glc/GlcA and Phe; these metabolites have specific variations in the Pd_2_Spm-induced metabolic signature. This correlation may reflect a glycolysis flux regulation, since Phe is known to decrease the activity of pyruvate kinase leading to glucose accumulation [60,61], which in turn may become available for UDP-Glc/GlcA synthesis. UDP-GlcA is involved in the synthesis of proteoglycans, which are extracellular matrix components that determine neuronal development and plasticity [50]. Therefore, this seems to be a clear pathway characterizing the response to the palladium agent. The additional NADH/Ado correlation is suggestive of deviations in the metabolism of purines [62], probably enhancing Ino synthesis (which is increased in the Pd_2_Spm-treated group) for a higher neuroprotective ability [46].

### 3.2. Metabolic Impact of Drugs on the Liver

The metabolic profile of liver is less affected by the drugs presently under study, compared to the brain, and similar qualitative effects are noted for the two drugs, although with distinct variation significance. GSH, His and Tyr are all increased more significantly upon cDDP administration. A Tyr increase has been usually associated with hepatic injury [63,64], whereas the change in His is not of straightforward interpretation at this stage. Interestingly, the increase in the liver GSH contradicts previous reports of GSH depletion in this organ, upon cDDP administration [18,65,66]. This indicates that GSH levels may be significantly time-dependent, also varying with the type of animal model, timings of post-treatment animal analysis and drug dosage/protocols, which tend to differ significantly between experimental studies. In any case, a variation in the liver GSH (not seen for Pd_2_Spm) reflects a significant hepatic antioxidant response to cDDP, consistently with the higher oxidative stress noted in the brain of the same animals when exposed to this Pt(II) agent. This GSH increase in the liver is consistent with increased levels of NADP^+^ (also not observed in the Pd_2_Spm group), a cofactor derived from the reduction in GSSG to GSH [67]. The above observations suggest a higher extension of the liver damage upon cDDP administration (hepatotoxicity, a known side effect of this drug). In addition, significantly increased levels of UDP-GlcNAc are observed for the cDDP-treated group, only an increasing tendency have been verified for Pd_2_Spm, suggesting an enhancement of the hexosamine biosynthetic pathway (HBP) and of protein glycosylation [68], a post-translational modification recently described in the regulation of the liver regeneration after partial hepatectomy [69]. We therefore suggest that the UDP-GlcNAc increase may be a measure of cell regeneration requirements in the liver. A lesser significant rise of UDP-GlcNAc in Pd_2_Spm-treated animals may reflect a lower degree of hepatic damage and hence less need for regeneration.

Pd_2_Spm exposure, contrary to cDDP, specifically diminishes the levels of the liver AMP and UMP, which evidences different nucleotides mechanisms for each of the drugs. This is corroborated by the significant pseudouridine decrease for the Pd_2_Spm treated samples, absent upon cDDP treatment, and indicative of how RNA pseudouridylation [70] may be an important issue in this context. The precise relationship of these observations with DNA and RNA metabolisms is unclear at this stage, although uridine metabolism is unveiled as of interest for future studies. Other metabolic response common to both drugs find higher relevance upon Pd_2_Spm treatment, such as the decreases in *O*-acetylcarnitine, Cho, Ado and malonate. Changes in *O*-acetylcarnitine and malonate suggest possible deviations in FA synthesis and β-oxidation, respectively, although overall FA levels remain practically unchanged. However, membrane metabolism in the liver seems to be distinct for different drug treatments, both inducing a Cho decrease (although more pronounced upon Pd_2_Spm) but in the case of cDDP, this is accompanied by GPC and PTE decreases. In addition, upon cDDP exposure there seems to be a shift towards Cho/TMA metabolism (as TMA is significantly increased), which may be mediated by gut microbiota [71,72] and possibly trigger hepatic pro-lipogenic and pro-inflammatory activities [73]. On the other hand, Pd_2_Spm administration only impacts weakly in all the above discussed metabolites (GPC and TMA show increasing tendencies only, whereas PTE hardly changes).

A positive correlation between malonate and ALC (both significantly decreased by drug exposures) was found in the three groups and reinforces an underlying regulation between FA synthesis and β-oxidation, respectively, in liver [74]. The concomitant malonate and ALC decreases upon either treatment may reflect the use of malonyl-CoA (synthesized from malonate by malonyl-CoA synthetase) to promote FA synthesis, while also having the ability of inhibiting carnitine acyltransferase I to avoid the futile FA synthesis/degradation cycle [74]. The untreated group is also characterized by positive correlations between GSH/UDP-GlcNAc and AMP/Cho. Interestingly, UDP-GlcNAc levels have been described to respond to oxidative stress, through the *O*-GlcNAcylation of enzymes (such as phosphofructokinase 1 and glucose-6-phosphate dehydrogenase), inhibiting glycolysis and prompting the flux of other pathways, such as the pentose phosphate pathway (PPP) and HBP [75]. The AMP/Cho relationship could indicate a possible effect of Cho altering mitochondrial lipid metabolism, through the activation of AMP-activated protein kinase [76]. However, this would require specific measurements of lipid metabolism, to be investigated further.

Exposure to cDDP unveils an additional correlation of malonate with Cho (besides ALC), consistent with Cho affecting lipid metabolism, not only through AMP-activated protein kinase (as suggested for controls), but also through downregulation of FA synthase [77]. Moreover, an interplay of GSH with NADP^+^ and UMP supports the involvement of nucleotides in antioxidant mechanisms, while UDP-GlcNAc/adenosine (−) may reflect the use of adenosine in ATP synthesis to support UDP-GlcNAc synthesis via HBP. Remarkably, Pd_2_Spm treatment retains FA metabolism regulation through malonate and ALC, with only an additional suggestion of GSH levels depending on gut microflora-mediated TMA, which is an interesting relationship to investigate further.

### 3.3. Inter-Organ Metabolic Relationships within the Liver-Brain Axis

The most interesting observation is that the effect of either drug on inter-organ correlation maps is to drastically simplify them, however, this may simply be the result of biochemical dispersion with metabolites serving several biochemical ends and thus decreasing the number of clear inter-metabolite correlations (to the point that a lower r threshold of 0.80 had to be used, compared to 0.90 for intra-organ correlations). However, without attempting to interpret the interesting but complex brain/liver correlation map, descriptive of untreated animals, it is worth noting that cDDP treatment produces a complete set of new inter-organ correlations, clearly relating the modulation of the brain BCAA (levels unchanged upon cDDP treatment) with higher contents of liver His and TMA. Furthermore, liver UMP (and NADP^+^) levels seem to directly correlate to the brain UMP levels, which illustrates the expected liver-brain axis process of supplying nucleotide pools to the brain, through de novo nucleoside synthesis in liver and their transport to the brain [78]. Hence, the above observations illustrate a clear cDDP-related metabolic relationship between the liver, brain and gut microflora (viewed by TMA), to be further investigated. In contrast, treatment with Pd_2_Spm, which involved marked increase in the brain BCAA, does not highlight such marker in the simple liver-brain correlation map. Rather, Pd_2_Spm induces new liver-brain relationships, compared to untreated animals, showing that the increased GSH levels in the brain seem to depend on the use of AMP in the liver (which is decreased by Pd_2_Spm), possibly indicating its use in ATP synthesis to support the de novo synthesis of GSH in astrocytes (by GCL and GSS) for maintenance of the redox homeostasis in the brain [41]. On the other hand, hepatic GSH levels (unchanged by Pd_2_Spm treatment) seem to be related with the glycerol levels in the brain, possibly reflecting a role of the brain lipid metabolism in modulating liver oxidative stress, in tandem with a possible regulation of hepatic glycosylation processes (unchanged levels of liver UDP-GlCNAc).

## 4. Materials and Methods

### 4.1. Ethical Considerations and Animal Procedures

Animals handling and care procedures were carried out in full compliance with the Portuguese (Decreto-Lei Nº. 113/2013) and European (Directive 2010/63/EU) legislation for the protection of animals used for scientific purposes and with the recommendations stated in the Guide for Care and Use of Laboratory Animals of the National Institutes of Health (NIH). The protocol followed in this study was approved by the Ethics Committee for Animal Experimentation of the Faculty of Pharmacy of the University of Porto, Porto, Portugal (Permit Number: 25-10-2015), and by the Ethics Committee and the Organ Responsible for the Welfare of Animals of ICBAS-UP, Porto, Portugal (Permit number 134/2015). All work followed The Animal Research: Reporting of In Vivo Experiments (ARRIVE) guidelines [79].

### 4.2. Drugs Administration and Tissue Collection

Cisplatin (*cis*-dichlorodiammine platinum (II) or cDDP, 99.9%), potassium tetrachloropalladate (II) (K_2_PdCl_4_, 98%), and spermine (*N*,*N*′-bis(3-aminopropyl)-1,4-diaminobutane, 99%) were obtained from Sigma-Aldrich Chemical S.A. (Sintra, Portugal). All reagents were of analytical grade. Euthasol^®^ solution (400 mg/mL pentobarbital sodium) was purchased from Le Vet (Oudewater, Utrecht, The Netherlands). Pd_2_Spm was synthesized according to previously reported procedures [80,81].

**Figure A1:**
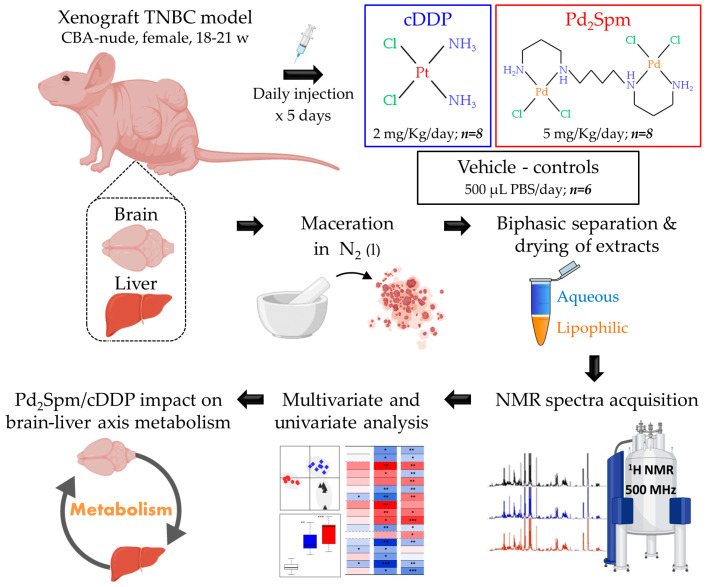
Experimental design workflow illustrating the animals’ exposure to the metal-drugs, cDDP (2 mg/kg/day) or Pd_2_Spm (5 mg/kg/day), and vehicle solution (phosphate-buffered saline, PBS), followed by brain and liver excision and extracts preparation using a mechanical maceration in liquid nitrogen (N_2_) and a biphasic separation with MeOH/H_2_O/CDCl_3_, and, ultimately, the NMR spectra acquisition and chemometric analysis. Figure created with BioRender.com [90].

Figure A1 (Appendix A) represents the whole workflow of this work. Female CBA nude (N:NIH(S)II-nu/nu) mice were purchased from i3S Animal Facility (Porto, Portugal) and acclimatized at least for 1 week at the ICBAS-UP Rodent Animal House Facility (Porto, Portugal), in a specific-pathogen-free (SPF) environment, as follows: housed in groups of 3 individually ventilated cages with enrichment material (corncob bedding, paper roll tube, and one large sheet of tissue paper for nesting), with ad libitum access to water and standard pellet food, under controlled 12 h light/dark cycles (lights on at 7:00 a.m.), temperature (22 ± 2 °C), and humidity (50 ± 10%). At 14 to 17-weeks-old, the animals were subcutaneously implanted in the left flank with triple-negative breast cancer (TNBC) MDA-MB-231 cells (25G needle, 5 × 10^6^ cells in 150 µL of PBS; according to procedures previously described [7]). At day 25 post-implantation (volume of tumors ~250 mm^3^), the mice were randomly allocated into three groups (8 animals per group) to receive the treatment with either (i) vehicle (phosphate-buffered saline, PBS)-controls, (ii) cDDP (2 mg/kg/day), or (iii) Pd_2_Spm (5 mg/kg/day), via intraperitoneal injection, during five consecutive days. The animals’ welfare was guaranteed by monitoring their physical activity, body weight, and measurement of tumor growth (criteria used to verify possible indicators of animals’ pain/distress related to disease progression as a humane endpoint). At day 28 post-implantation, two control animals developed ulcerated tumors, so these mice needed to be prematurely euthanized and excluded from the study. The final group sizes were *n* = 6 for controls, and *n* = 8 for each treated group. At day 39 post-implantation, animals were euthanized with isoflurane, followed by cardiac punction, the whole brain and liver were excised, washed in PBS, and weighted (average brain weights: 0.45 ± 0.027 g for controls, 0.44 ± 0.03 g for cDDP-treated group and 0.45 ± 0.028 g for Pd_2_Spm-treated group; average liver weights: 1.5 ± 0.12 g for controls and 1.6 ± 0.11 g for both treated groups).

### 4.3. Brain and Liver Extraction

The frontal cortex of the brain and half of the liver (comprising median and left lobes) from mice were excised and weighted (0.099 ± 0.023 g for controls, 0.078 ± 0.011 g for cDDP-treated group, and 0.098 ± 0.019 g for Pd_2_Spm-treated group), maintaining the samples in dry ice. Each tissue sample was ground to a fine powder, using a pestle and mortar, in liquid N_2_ [82,83,84] and sample extracts were obtained by a biphasic separation method of methanol/chloroform/water (2.0/2.0/1.0), as described elsewhere [85]. The aqueous and lipophilic extracts (upper and bottom phases, respectively) were collected separately, dried in vacuum and under N_2_, respectively, and stored at −80 °C until analysis.

### 4.4. NMR Spectroscopy

Prior to NMR spectra acquisition, the dried extracts were suspended in 650 µL of 100 mM sodium phosphate buffer (pH 7.4) in D_2_O containing 0.25% 3-(trimethylsilyl)-propionic-2,2,3,3-d4 acid (TSP) or in 650 µL of CDCl_3_ containing 0.03% tetramethylsilane (TMS), for polar or lipophilic extracts, respectively. NMR spectra were acquired on a Bruker AVANCE III HD spectrometer (Bruker, Ettlingen, Germany), operating at 500.13 MHz for proton, at 298 K. The unidimensional spectra were acquired using the “noesypr1d” and “zg” pulse sequences (Bruker library), for aqueous and lipophilic extracts, respectively, using acquisition parameters described elsewhere [14]. The spectra were manually phase and baseline corrected and internally calibrated to TSP/TMS signals at δ 0.0 ppm. Bidimensional NMR spectra, homonuclear total correlation (TOCSY) and heteronuclear single-quantum correlation (HSQC), were acquired for selected samples to aid peak assignment, which was also based on the literature and databases, such as Bruker BIOREFCODE (spectral database of AMIX-viewer 3.9.14, Bruker Biospin, Rheinstetten, Germany), human metabolome database (HMDB) [86] and Chenomx NMR Suite (Chenomx Inc., Edmonton, AB, Canada).

### 4.5. Data Processing and Statistical Analysis

Unidimensional (1D) NMR spectra were converted into matrices (AMIX-viewer 3.9.14, Bruker Bio-spin, Rheinstetten, Germany), including a spectral width of δ 0.5 to 9.5 ppm and excluding the spectral regions of water suppression (δ 4.6–5.1 ppm) and methanol contamination (singlet at δ 3.36 ppm) for aqueous extracts, and residual signals of water (δ 1.5–1.8 ppm) and both chloroform and corresponding satellites peaks (δ 7.0–7.5 ppm) for lipophilic extracts. Spectra were aligned by recursive segment-wise peak alignment (RSPA) to minimize chemical shift variations (Matlab 8.3.0, The MathWorks Inc., Natick, MA, USA), and normalized to total spectral area to minimize the potential effects of different samples weight. Multivariate analysis was carried out using unsupervised and supervised methods, namely principal component analysis (PCA) and partial least-squares discriminant analysis (PLS-DA), considering two latent variables, upon unit variance (UV) scaling (SIMCA-P 11.5; Umetrics, Umeå, Sweden). PLS-DA models were considered statistically robust for predictive power (Q^2^) values higher than 0.5. PLS-DA loadings were back-transformed, multiplying each variable by its standard deviation, and colored according to variable importance to the projection (VIP) (Matlab 8.3.0, The MathWorks Inc., Natick, MA, USA). The resonances relevant for class separation, identified in PLS-DA loading plots and confirmed by visual inspection of spectra, were integrated (Amix-multi integrate tool 3.9.14, Bruker BioSpin, Rheinstetten, Germany), normalized, and variations assessed by univariate analysis, combining the calculation of effect-size (ES) [87] and statistical significance (Shapiro–Wilk test to assess data normality, Student’s *t*-test or Wilcoxon test for normally distributed or non-normally distributed data, respectively) (R-statistical software). After spectral confirmation of changes, metabolites levels were considered to vary significantly for |ES| > ES error and *p* < 0.05, and *p*-values were corrected by False discovery rate (FDR), with the basis on the Benjamini and Hochberg method [88]. Significant deviations were putatively interpreted with basis on information comprised in the Kyoto Encyclopedia of Genes and Genomes (KEGG) database [89]. Spearman’s rank correlations were carried out (R-statistical software) for the spectra of aqueous extracts alone, due to their higher resolution. Only correlations with a significance of *p* < 0.05 were considered, with 0.80 and 0.90 as minimum correlation coefficient (r) thresholds for intra- and inter-organ correlations, respectively.

## 5. Conclusions

This paper reports, for the first time to our knowledge, a detailed untargeted metabolic characterization of the brain and liver of xenograft mice of a TNBC model subjected to treatment with either cDDP (as a reference drug) or Pd_2_Spm (as a potential new anticancer drug). NMR metabolomics measured the effects of each drug on both polar and lipophilic tissue extracts and the results revealed that their impact was unequivocally stronger on brain than on liver, particularly for Pd_2_Spm. Importantly, neither cDDP nor Pd_2_Spm exposure seems to induce neuronal damage or inflammation, at least considering the known metabolic indicators.

Compared to cDDP, Pd_2_Spm induced a more effective brain response to oxidative stress, with GSH levels maintained high with the aid of cystathionine to reinforce GSH biosynthesis. Larger pools of brain BCAA were found, possibly to ensure a suitable regulation of essential bioactive brain metabolites such as Glu, GABA and Gln. Nucleotides and nucleosides seemed to play important roles in inducing a more favorable adaptive response of the brain to Pd_2_Spm, an apparent enhanced role of Ino in anti-inflammatory and antioxidant mechanisms and an interesting interplay of uridine-derived compounds (which will require further investigation in future studies). In addition, cholesterol, cell membrane and proteoglycan metabolisms showed stronger responses to Pd_2_Spm treatment as compared to cisplatin, which is probably indicative of a higher adaptability of the cell membrane characteristics and extracellular function.

Considering the much less affected metabolism of liver, cDDP showed a slightly stronger effect on this organ than Pd_2_Spm, with a suggestion of a higher extension of liver damage (as expected) and a possible enhanced involvement of UDP-GlcNAc in liver regeneration processes. Uridine metabolism was again identified as particularly important in the response of liver to either drug.

Intra- and inter-organ correlation studies added to the biochemical information already gathered, although their complex patterns could only be putatively and subjectively interpreted, based on metabolic data alone. However, it could be suggested that cDDP treatment seems to achieve brain BCAA regulation at the expense of liver His and TMA, whereas liver and brain UMP were found to be directly correlated. In addition, Pd_2_Spm treatment was confirmed to involve strong antioxidant protective mechanisms, that may be partly due to the presence of the spermine polyamine (a known biogenic antioxidant). In turn, brain GSH levels seem to be maintained at the expense of liver AMP, whereas hepatic GSH appears to be determined, at least in part, by brain glycerol (and possibly lipid metabolism), also suggested to help regulate liver protein glycosylation.

These results illustrate how informative untargeted metabolomics can be for evaluating the impact of anticancer drugs on multiple organs. Still, a limitation of this work (which will find scope in future studies) is the absence of catalytic activities of specific enzymes to confirm up- or downregulation of specific pathways. Furthermore, the effects revealed for Pd_2_Spm on brain and liver seem significantly beneficial compared to those exhibited by cDDP treatment, probably reflecting fewer secondary effects, in spite of its higher anti-cancer ability. This renders the palladium agent a promising candidate for a possible replacement of cDDP, or, more probably, for combined cDDP-Pd_2_Spm therapeutic protocols.

## Data Availability

The data presented in this study are openly available in Metabolomics Workbench: An international repository for metabolomics data and metadata, metabolite standards, protocols, tutorials and training, and analysis tools (2016), using the website https://www.metabolomicsworkbench.org, (accessed on 29 September 2022) and both http://dx.doi.org/10.21228/M8S124 (accessed on 19 September 2022) and reference numbers ST002290 and ST002293 for aqueous and lipophilic extracts of brain, respectively, as well as ST002294 and ST002295 for aqueous and lipophilic extracts of liver, respectively.

## Supplementary Materials

The supporting information can be downloaded at: https://www.mdpi.com/article/10.3390/ijms232213773/s1.
