# Supplementary material for: Effect of Pd2Spermine on Mice Brain-Liver Axis Metabolism Assessed by NMR Metabolomics
Source: Int J Mol Sci. 2022 Nov 9;23(22):13773. doi: 10.3390/ijms232213773 (PMC9693583; doi:10.3390/ijms232213773)
Supplement: Supplementary file 1 [file ijms-23-13773-s001.zip › ijms-2014735-supplementary.pdf]

International Journal of
Molecular Sciences

MDPI

Supplementary Materials

Effect of Pd_2Spermine on Mice Brain-Liver Axis Metabolism Assessed by NMR Metabolomics

Tatiana J. Carneiro [1], Martin Vojtek [2], Salomé Gonçalves-Monteiro [2], Ana L. M. Batista de Carvalho [3], Maria Paula M. Marques [3,4], Carmen Diniz [2] and Ana M. Gil [1,*]

1 Department of Chemistry and CICECO—Aveiro Institute of Materials, University of Aveiro, 3810-193 Aveiro, Portugal
2 LAQV/REQUIMTE, Laboratory of Pharmacology, Department of Drug Sciences, Faculty of Pharmacy, University of Porto, 4150-755 Porto, Portugal
3 Molecular Physical-Chemistry R&D Unit, Department of Chemistry, University of Coimbra, 3004-535 Coimbra, Portugal
4 Department of Life Sciences, Faculty of Science and Technology, University of Coimbra, 3000-456 Coimbra, Portugal
* Correspondence: agil@ua.pt; Tel.: +351-234370707

Figure S1. Average 500 MHz ^{1}H NMR spectra of lipophilic extracts of (**a**) brain, and (**b**) liver from the control (untreated) group of a CDX mouse model of TNBC.

Figure S2. Average 500 MHz ^{1}H NMR spectra of aqueous extracts of liver, from a CDX mouse model of TNBC, exposed to (**a**) vehicle solution (control group), (**b**) cDDP (2 mg/kg/day), and (**c**) Pd_2Spm (5 mg/kg/day).

Figure S3. (**a**) Spectral expansions (δ 8.2-9.6 ppm) of ^{1}H NMR spectra of aqueous extracts of CDX mice brain of controls, cDDP- and Pd_2Spm-treated mice and (**b**) NAD^+/NADH ratios for the same three animal groups.

Figure S4. Pairwise score scatter plots of PCA and PLS-DA models for ^{1}H NMR spectra of (**a**) aqueous and (**b**) lipophilic extracts of liver of CDX mice.

Table S1. Statistically significant ($|ES| >$ ES Error and *p*-value < 0.05) metabolite variations observed in the polar metabolomes of CDX mice brain and liver, compared to controls.

Table S2. Statistically significant ($|ES| >$ ES Error and *p*-value < 0.05) metabolite variations observed in the lipophilic metabolomes of CDX mice brain and liver, compared to controls.

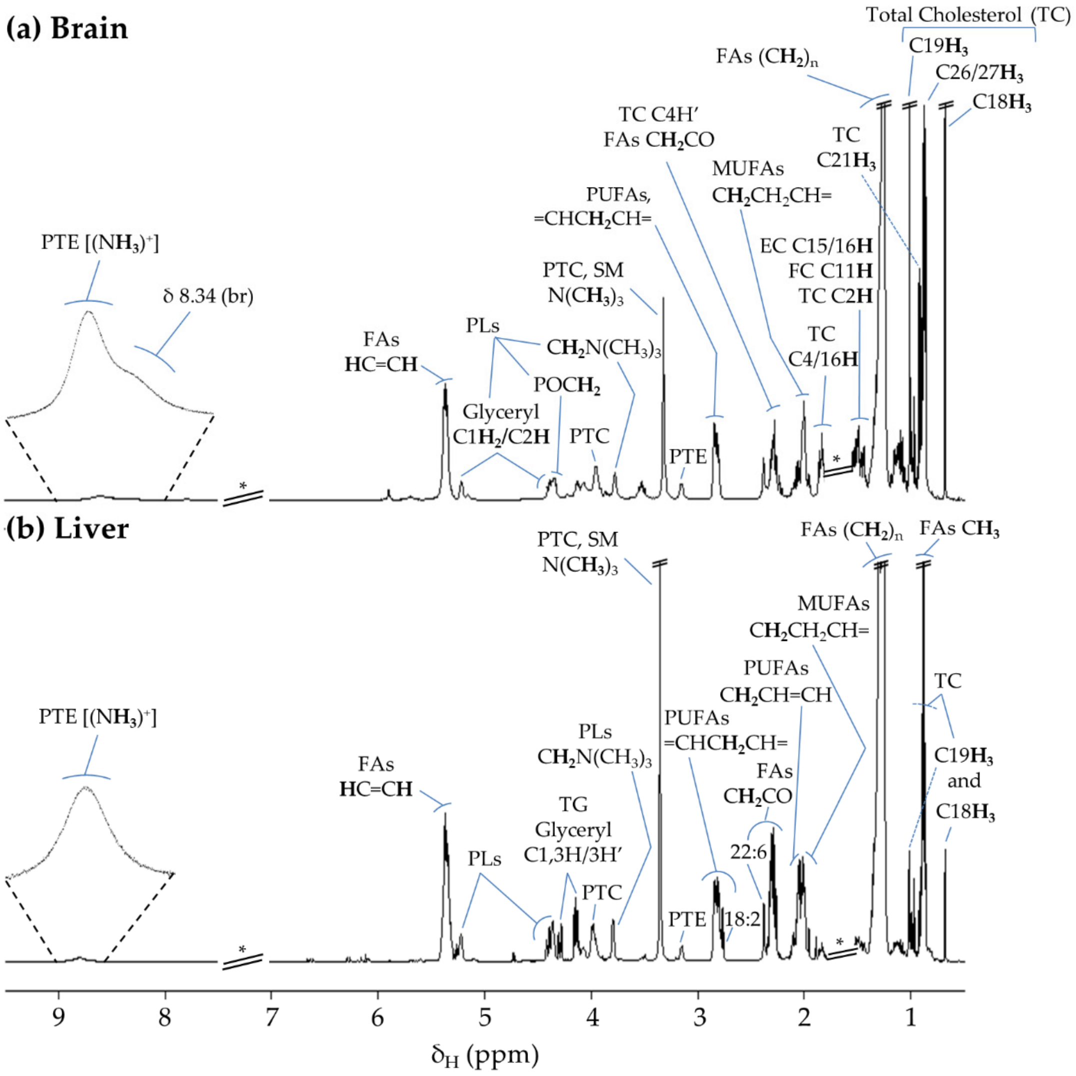

Figure S1. Average 500 MHz ^{1}H NMR spectra of lipophilic extracts of (**a**) brain, and (**b**) liver from the control (untreated) group of a CDX mouse model of TNBC. * Cut-off of residual water signal (δ 1.5–1.8 ppm) and $CDCl_3$ and corresponding satellites (δ 7.0–7.5 ppm), not considered for multivariate analysis. Abbreviations: EC, esterified cholesterol; FAs, fatty acids; FC, free cholesterol; MUFAs, monounsaturated fatty acids; PLs, phospholipids; PTC, phosphatidylcholine; PTE, phosphatidylethanolamine; PUFAs, polyunsaturated fatty acids; SM, sphingomyelin; TC, total cholesterol; TG, triacylglycerols; br, broad signal.

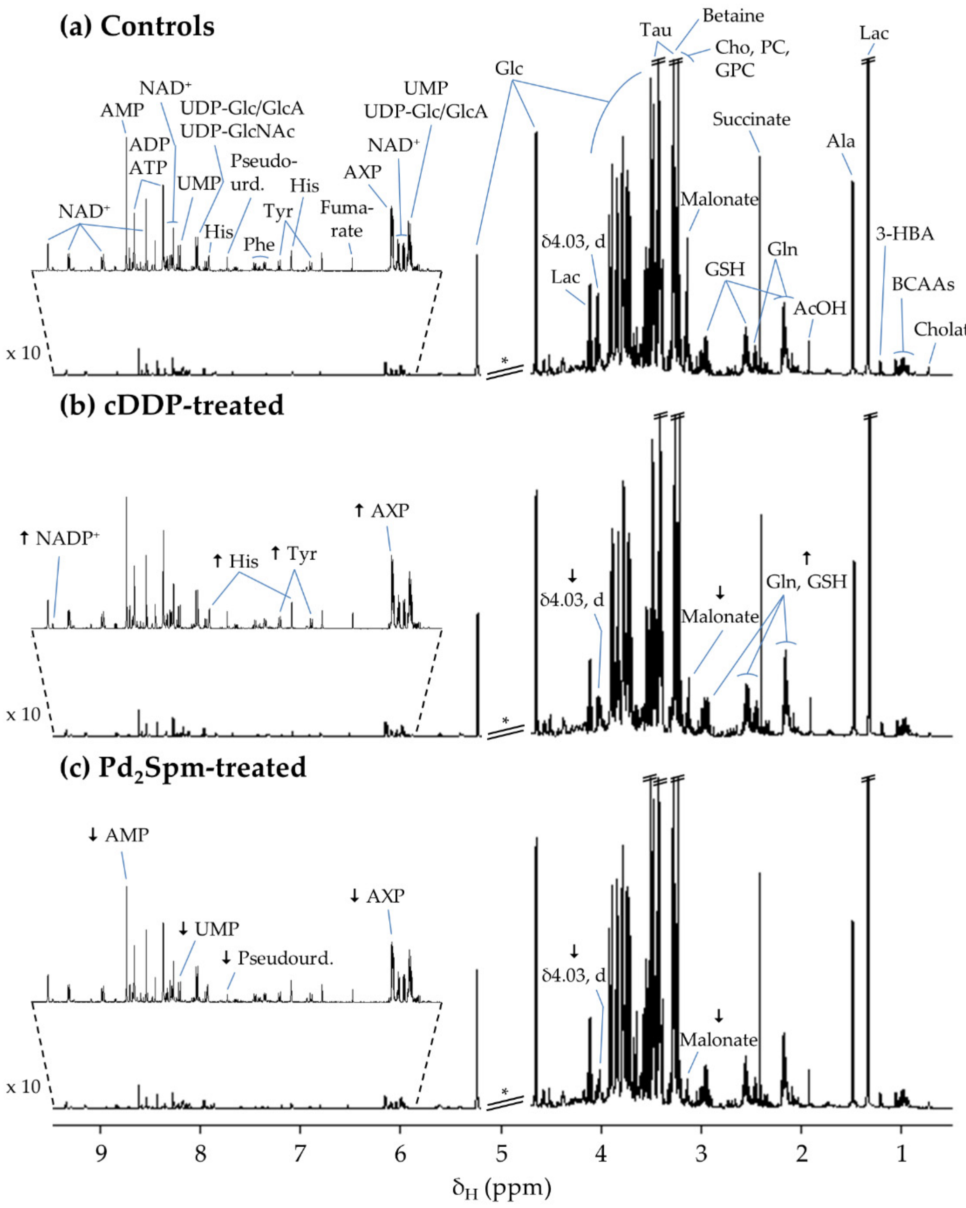

Figure S2. Average 500 MHz ^{1}H NMR spectra of aqueous extracts of liver, from a CDX mouse model of TNBC, exposed to (**a**) vehicle solution (control group), (**b**) cDDP (2 mg/kg/day), and (**c**) Pd_2Spm (5 mg/kg/day). * Cut-off of water suppression region (δ 4.6–5.1 ppm), not considered for multivariate analysis. The arrows in (**b**) and (**c**) identify visual metabolic variations found with qualitative inspection of spectra from each treated group compared to controls. Abbreviations: 3-letter code for amino acids; 3-HBA, 3-hydroxybutyrate;; AcOH, acetate; ADP, adenosine diphosphate; AMP, adenosine monophosphate; ATP, adenosine triphosphate; AXP, adenosine nucleotides AMP, ADP and ATP; BCAAs, branched-chain amino acids (ile, leu and val); Cho, choline; Glc, glucose; GPC, glycerophosphocholine; GSH, glutathione (reduced); Lac, lactate; NAD^+, nicotinamide adenine dinucleotide (oxidized); $NADP^+$, nicotinamide adenine dinucleotide phosphate (oxidized); PC, phosphocholine; Pseudourd., pseudouridine (Ψ, tentative assignment); Tau, taurine; UDP-Glc/GlcA, uridine diphosphate glucose/glucuronate; UDP-GlcNAc, uridine diphosphate *N*-acetylglucosamine; UMP, uridine monophosphate; d, doublet.

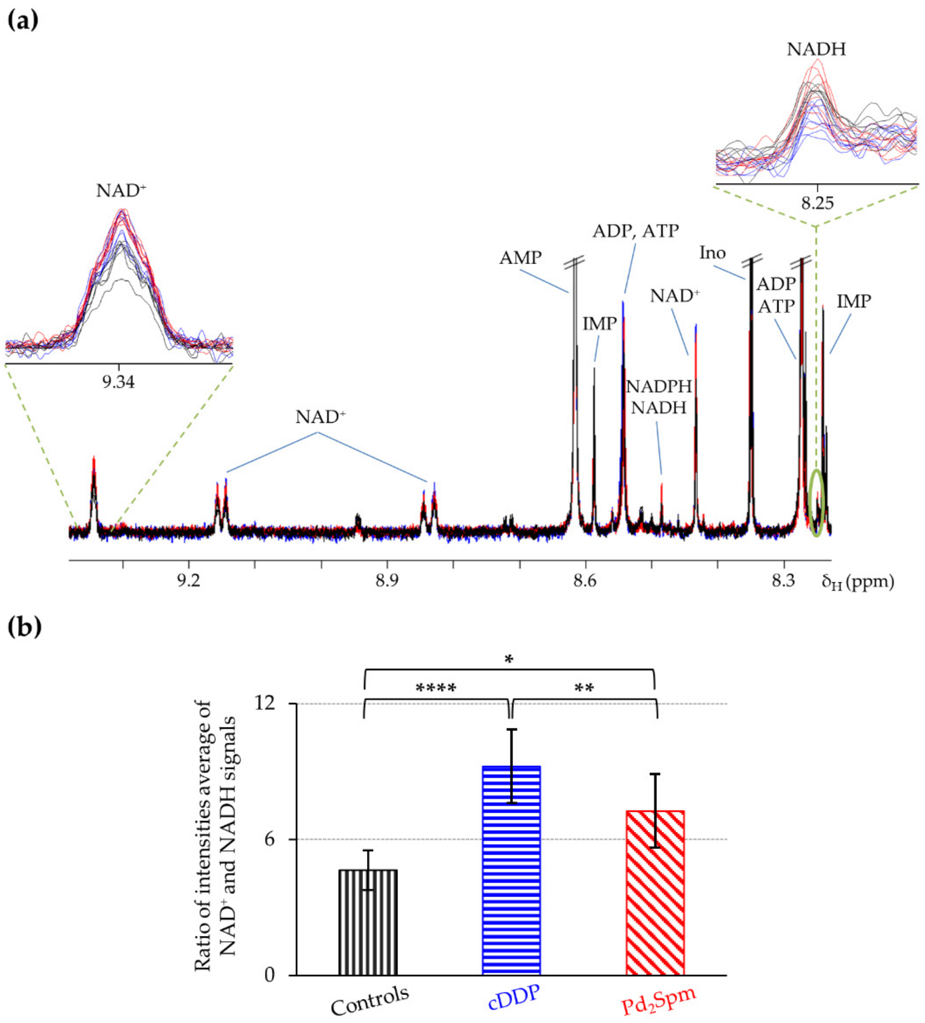

Figure S3. (**a**) Spectral expansions (δ 8.2-9.6 ppm) of the ^{1}H NMR spectra of aqueous extracts of CDX mice brain of controls (black trace), cDDP- (blue trace) and Pd2Spm-treated (red trace) mice and (**b**) NAD^+/NADH ratios for the same three animal groups; error bars indicate the respective standard deviation. * *p*-value < 5.0×10^{-2}; ** *p*-value < 1.0×10^{-2}; **** *p*-value < 1.0×10^{-4}.

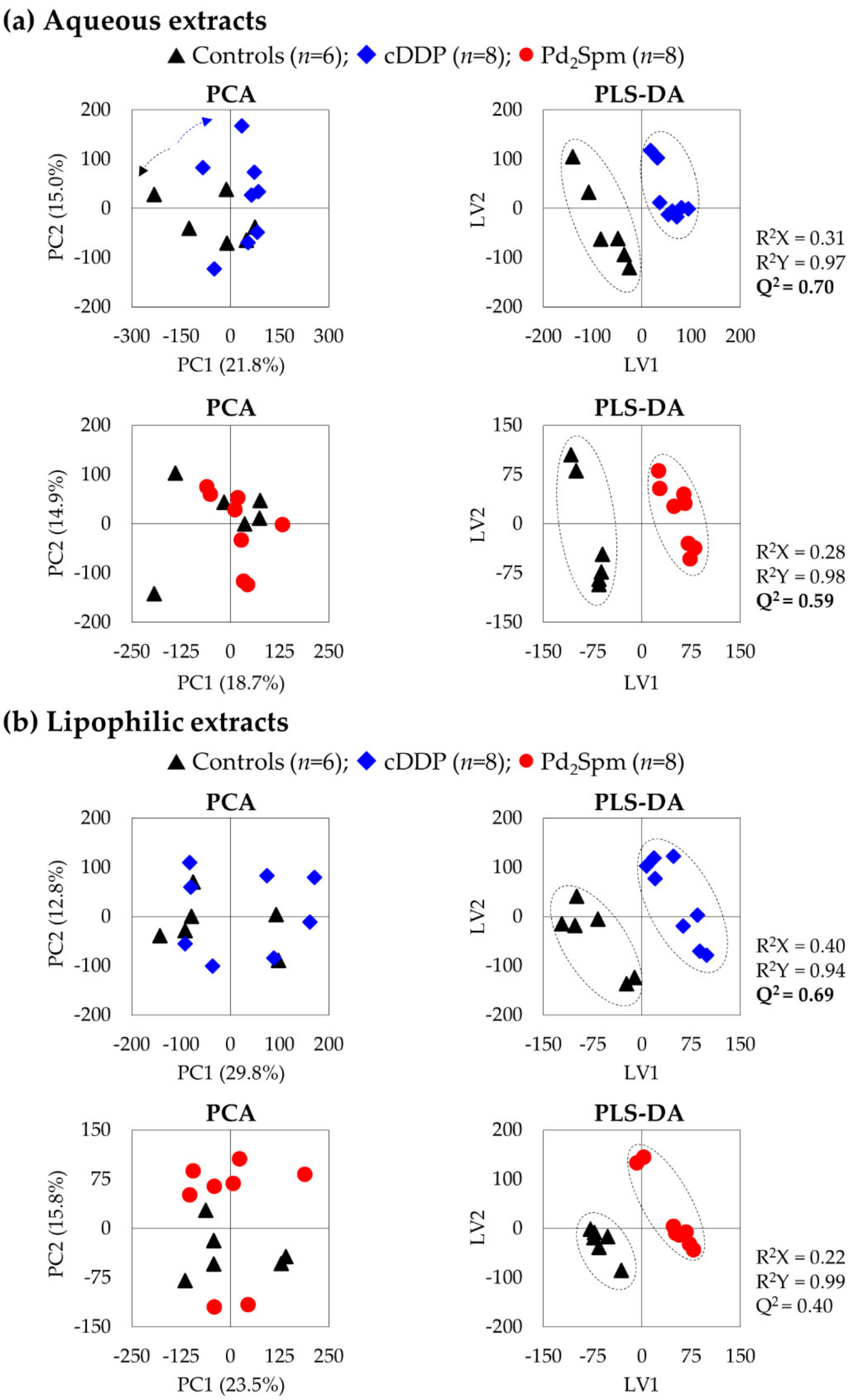

Figure S4. Pairwise score scatter plots of PCA and PLS-DA models for ^{1}H NMR spectra of (**a**) aqueous and (**b**) lipophilic extracts of liver of CDX mice (controls, black triangles, *n* = 6; cDDP-treated, blue diamonds, *n* = 8; Pd_2Spm-treated, red circles, *n* = 8). Validation parameters (R^2 and Q^2) are indicated for each PLS-DA model, with Q^2 values > 0.5 highlighted in bold indicating robust classes separation.

Table S1. Statistically significant (|ES| > ES Error and *p*-value < 0.05) metabolite variations observed in the polar metabolomes of CDX mice brain and liver, compared to controls. † Tentative assignment. ‡ Partial integration of resonance peak. [a] Metabolic variation statistically significant after False Discovery Rate (FDR) correction. Abbreviations: 3-letter code used for amino acids; 3-HIBA, 3-hydroxyisobutyrate; Ado, adenosine; AMP, adenosine monophosphate; Cho, choline; GPC, glycerophosphocholine; GSH, glutathione (reduced); IMP, inosine monophosphate; Ino, inosine; NAA, *N*-acetylaspartate; NAD^+/NADH, nicotinamide adenine dinucleotide (oxidized/reduced); $NADP^+$, nicotinamide adenine dinucleotide phosphate (oxidized); UDP-Glc/GlcA, uridine diphosphate glucose/glucuronate; UDP-GlcNAc, uridine diphosphate *N*-acetylglucosamine; UMP, uridine monophosphate; TMA, trimethylamine; s, singlet; d, doublet; dd, double doublet; t, triplet; m, multiplet.

		**cDDP *vs.* Controls**		**Pd_2Spm *vs.* Controls**	
**Metabolite**	**δ/Multiplicity**	**ES ± Error**	***p*-Value**	**ES ± Error**	***p*-Value**
**BRAIN**					
3-Aminoisobutyrate	1.18 (d)	1.3 ± 1.2	2.1×10^{-2} [a]	3.5 ± 1.7	5.9×10^{-5} [a]
3-HIBA	1.09 (d)	-	-	4.1 ± 1.8	2.1×10^{-5} [a]
Adenine	8.23 (s)	-	-	-1.5 ± 1.2	3.2×10^{-2}
Ado	8.349 (s)	-	-	-1.9 ± 1.3	6.7×10^{-4} [a]
AMP	8.60 (s)	-	-	-1.6 ± 1.2	1.3×10^{-2} [a]
Cholate †	0.73 (s)	1.2 ± 1.1	4.0×10^{-2}	4.7 ± 2.0	3.2×10^{-6} [a]
Creatine	3.04 (s)	-	-	-1.5 ± 1.2	1.3×10^{-2} [a]
Cystathionine †	2.74 (d) ‡	-	-	3.5 ± 1.7	2.1×10^{-5} [a]
Glycerol	3.55 (m)	-1.7 ± 1.2	1.3×10^{-3} [a]	-	-
GSH	2.96 (m)	1.9 ± 1.3	4.1×10^{-3} [a]	2.1 ± 1.3	2.9×10^{-3} [a]
Ile	0.94 (t)	-	-	2.1 ± 1.3	1.6×10^{-3} [a]
IMP	8.23 (s)	-	-	5.8 ± 2.4	5.4×10^{-6} [a]
Ino	8.35 (s)	-	-	2.1 ± 1.3	1.3×10^{-3} [a]
Leu	0.96 (t)	-	-	2.4 ± 1.4	6.4×10^{-4} [a]
Lys	1.73 (m)	-	-	6.0 ± 2.5	5.9×10^{-8} [a]
Malate †	2.66 (dd)	-1.3 ± 1.2	2.3×10^{-2} [a]	-1.3 ± 1.2	2.5×10^{-2} [a]
NAA	2.02 (s)	-	-	-1.4 ± 1.2	4.8×10^{-2}
NAD^+	9.34 (s)	2.1 ± 1.3	1.3×10^{-3} [a]	3.6 ± 1.7	6.7×10^{-4} [a]
NADH	8.25 (s)	-3.5 ± 1.7	1.9×10^{-5} [a]	-	-
Pantothenate	0.74 (s)	-	-	5.3 ± 2.2	2.6×10^{-7} [a]
Phe	7.33 (m)	-	-	2.9 ± 1.5	1.3×10^{-4} [a]
Pseudouridine †	7.68 (s)	-	-	1.3 ± 1.2	2.1×10^{-2} [a]
Succinate	2.41 (s)	-	-	-1.5 ± 1.2	1.2×10^{-2} [a]
UDP-Glc/GlcA	5.97 (m)	-	-	1.6 ± 1.2	7.8×10^{-3} [a]
UMP	8.12 (d)	-1.6 ± 1.2	6.9×10^{-3} [a]	-1.5 ± 1.2	9.9×10^{-3} [a]
Val	1.05 (d)	-	-	3.3 ± 1.6	3.9×10^{-5} [a]
U1	1.46 (d)	1.4 ± 1.2	1.4×10^{-2} [a]	5.4 ± 2.3	2.7×10^{-7} [a]

U2	2.87 (s)	-	-	7.7 ± 3.0	4.3×10^{-9} [a]
U3	2.98 (d)	-	-	-1.9 ± 1.3	3.3×10^{-3} [a]
U4	3.33 (s)	-	-	-1.4 ± 1.2	2.0×10^{-2} [a]
**LIVER**					
O-Acetylcarnitine [†]	3.18 (s)	-1.8 ± 1.2	4.8×10^{-3} [a]	-4.0 ± 1.8	7.3×10^{-6} [a]
AMP	8.60 (s)	-	-	-2.2 ± 1.3	2.7×10^{-3} [a]
Ado	6.08 (d)	-2.3 ± 1.4	1.4×10^{-3} [a]	-3.1 ± 1.6	3.3×10^{-4} [a]
Cho	3.20 (s)	-2.3 ± 1.4	8.7×10^{-4} [a]	-3.8 ± 1.8	8.0×10^{-6} [a]
GPC	3.23 (s)	-1.4 ± 1.2	1.8×10^{-2} [a]	-	-
GSH	2.17 (m)	1.9 ± 1.3	4.4×10^{-3} [a]	-	-
His	7.08 (s)	2.3 ± 1.4	7.9×10^{-4} [a]	-	-
Malonate	3.13 (s)	-1.9 ± 1.3	3.1×10^{-3} [a]	-4.2 ± 1.9	6.7×10^{-4} [a]
$NADP^+$	9.30 (s)	1.3 ± 1.2	2.5×10^{-2}	-	-
Pseudouridine [†]	7.68 (s)	-	-	-1.8 ± 1.3	1.4×10^{-2} [a]
TMA	2.89 (s)	1.8 ± 1.3	8.0×10^{-3} [a]	-	-
Tyr	6.90 (d)	1.2 ± 1.2	5.0×10^{-2}	-	-
UDP-GlcNAc	5.52 (dd)	1.5 ± 1.2	3.2×10^{-2} [a]	-	-
UMP	8.12 (d)	-	-	-1.6 ± 1.2	1.3×10^{-2} [a]
U5	3.13 (s)	-1.7 ± 1.2	5.9×10^{-3} [a]	-4.6 ± 2.0	5.1×10^{-6} [a]
U6	3.33 (s) [‡]	1.2 ± 1.2	3.2×10^{-2} [a]	-	-
U7	4.03 (d)	-1.6 ± 1.2	8.5×10^{-3} [a]	-3.7 ± 1.7	3.4×10^{-5} [a]

Table S2. Statistically significant (|ES| > ES Error and p-value < 0.05) metabolite variations observed in the lipophilic metabolomes of CDX mice brain and liver, compared to controls. † Tentative assignment. ‡ Partial integration of resonance peak. [a] Metabolic variation statistically significant after False Discovery Rate (FDR) correction. Abbreviations: FAs, fatty acids; PLs, phospholipids; PTC, phosphatidylcholine; PTE, phosphatidylethanolamine; PUFAs, polyunsaturated fatty acids; TG, triacylglycerols; s, singlet; d, doublet; t, triplet; q, quartet; br, broad signal.

		cDDP *vs.* Controls		Pd_2Spm *vs.* Controls	
**Metabolite**	**δ/Multiplicity**	**ES ± Error**	***p*-Value**	**ES ± Error**	***p*-Value**
**BRAIN**					
Lathosterol $C18H_3$ †	0.54 (d)	-	-	-2.3 ± 1.4	1.4×10^{-2} [a]
FA $(CH_2)_n$	1.25 (br)	-	-	3.2 ± 1.7	8.5×10^{-3} [a]
PUFAs (ω3) CH_3	0.98 (t)	-	-	-2.2 ± 1.4	2.4×10^{-3} [a]
18:2 $=CHCH_2CH=$	2.77 (t)	-1.4 ± 1.2	4.6×10^{-2}	-3.0 ± 1.6	2.5×10^{-3} [a]
TG Glyceryl C2H †	5.29 (t)	-	-	-2.4 ± 1.4	1.1×10^{-2} [a]
PL $CH_2N(CH_3)_3$	3.75 (br)	-	-	3.1 ± 1.6	7.1×10^{-3} [a]
PL †	8.36 (br)	-	-	1.8 ± 1.3	3.1×10^{-3} [a]
PTC Glyceryl $C3H_2$ †	3.90 (br)	-	-	2.1 ± 1.4	1.1×10^{-2} [a]
PTE $(NH_3)^+$	8.80 (br)	-	-	-2.3 ± 1.4	1.3×10^{-3} [a]
U1	2.20 (q)	-1.4 ± 1.2	4.4×10^{-2}	-1.9 ± 1.3	2.5×10^{-2} [a]
U2	3.84 (d) ‡	-1.6 ± 2.3	1.3×10^{-2} [a]	-4.1 ± 1.9	1.6×10^{-4} [a]
**LIVER**					
PUFAs $CH_2CH=$	2.04 (q)	-1.3 ± 1.2	2.9×10^{-2}	-	-
PTE $(NH_3)^+$	8.80 (br)	-1.7 ± 1.2	6.7×10^{-3} [a]	-	-
U3	1.015 (s)	-	-	-1.5 ± 1.2	3.1×10^{-2}
U4	2.34 (br)	-	-	-1.3 ± 1.2	4.3×10^{-2}
U5	3.64 (s)	1.5 ± 1.2	1.3×10^{-2}	-	-
U6	3.88 (s)	-	-	2.0 ± 1.3	2.1×10^{-3} [a]
